# Laparoscopic-Assisted Enterolithotomy for Recurrent Gallstone Ileus: A Case Report

**DOI:** 10.7759/cureus.74123

**Published:** 2024-11-20

**Authors:** Kevin Vallejo, Claudia Morales, Alexa Denton, Deep Vakil, Lucia Castro Hernandez, Charles Vallejo, Fazaldin Moghul, Christopher Seaver

**Affiliations:** 1 College of Medicine, Florida International University, Herbert Wertheim College of Medicine, Miami, USA; 2 Department of Surgery, Memorial Healthcare System, Hollywood, USA; 3 Internal Medicine, Florida Atlantic University, Boca Raton, USA; 4 Department of General Surgery, Memorial Healthcare System, Hollywood, USA

**Keywords:** case report, entero-biliary fistulae, gallstone ileus, gallstones complication, gastrointestinal ileus, gastrointestinal obstruction, laparoscopic-assisted enterolithotomy

## Abstract

Gallstone ileus is the mechanical obstruction of the bowel due to gallstone impaction. It forms when a fistula is created between the gallbladder and the gastrointestinal tract, which can result in small bowel obstruction. Its surgical management ranges from enterolithotomy, cholecystectomy, and fistula closure performed together (one-stage) or performed separately (two-stage), while some patients undergo simple enterolithotomy. Emergency surgery with open enterolithotomy, with or without biliary tract surgery, has been replaced by laparoscopic-assisted enterolithotomy as a safer and more rapid procedure. This report is of a 68-year-old woman treated with laparoscopic-assisted enterolithotomy for gallstone ileus which recurred.

A 68-year-old woman with type 2 diabetes mellitus, hypertension, breast cancer, and end-stage renal disease on hemodialysis presented with a gallstone ileus and was surgically managed with successful laparoscopic-assisted enterolithotomy. Seven days after the initial surgery, she again presented with gallstone ileus requiring reoperation. A repeat laparoscopic-assisted enterolithotomy was performed with no complications and full resolution of her symptoms.

Operative management of gallstone ileus and subsequent recurrence continues to be highly debated. With no randomized studies and limited data, there is no current gold standard surgical procedure for either setting. Simple laparoscopic-assisted enterolithotomy is the favored surgical technique as it is associated with decreased morbidity, mortality, operative time, and complications. This report demonstrates that a CT scan is crucial in differentiating recurrent gallstone ileus from postoperative ileus, with a repeat laparoscopic-assisted enterolithotomy providing a safe and effective treatment option. Moreover, patient follow up is essential for monitoring symptom resolution.

## Introduction

Gallstone ileus accounts for 1% of all small bowel obstruction (SBO) cases [1]. It is a known, but infrequent, complication of cholelithiasis. The inflammation and the pressure effect of the offending gallstone cause erosion through the gallbladder wall, leading to the formation of a fistula between the gallbladder and an adjacent portion of the gastrointestinal tract, thus allowing the passage of the stone [2]. Gallstone ileus cases are seen in higher frequencies in the elderly population, causing about 25% of SBOs over the age of 65, with studies also indicating a 1:7 male-to-female ratio in occurrence [3,4]. The clinical signs and symptoms of gallstone ileus are usually non-specific but typically include abdominal pain, nausea, vomiting, and distension. Many imaging modalities are available but computed tomography (CT) is favored as the gold standard for diagnosis [1,5]. Surgery is the mainstay of treatment for gallstone ileus. The current surgical procedures are simple enterolithotomy; enterolithotomy, cholecystectomy, and fistula closure (one-stage procedure); and enterolithotomy with interval cholecystectomy and fistula closure (two-stage procedure) [3]. The rate of recurrence for gallstone ileus is reported to be less than 5%, with most cases occurring within six weeks from the initial episode [4,6]. Given its small rate of recurrence, studies on the best course of treatment are extremely limited. This report describes a 68-year-old woman treated with laparoscopic-assisted enterolithotomy for gallstone ileus with its recurrence seven days later, requiring repeat laparoscopic-assisted enterolithotomy. This report has been reported in line with the Surgical CAse REport (SCARE) criteria [7]. 

## Case presentation

**Figure 1:**
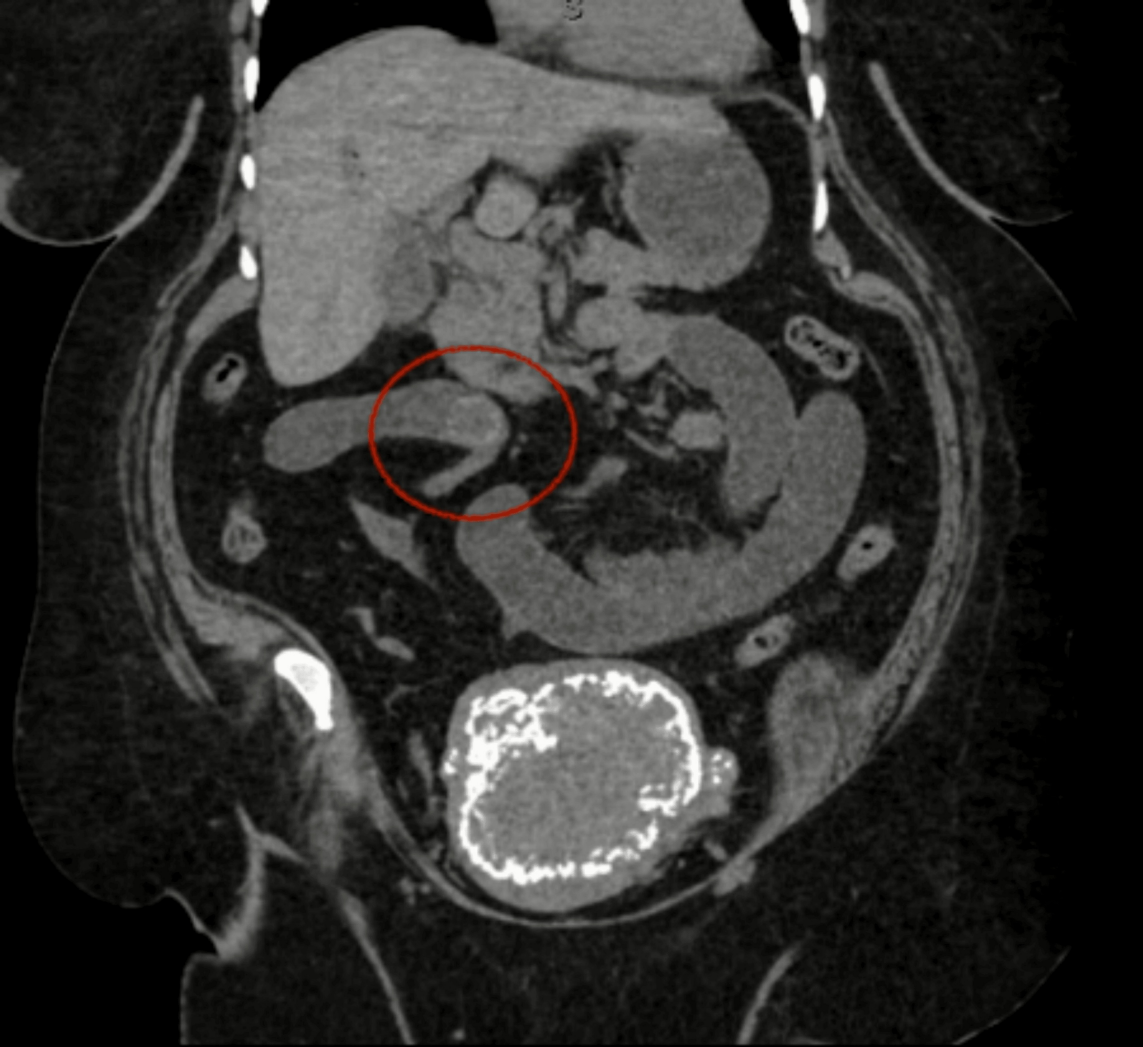
CT demonstrating a stone in the small bowel (circled in red)

**Figure 2:**
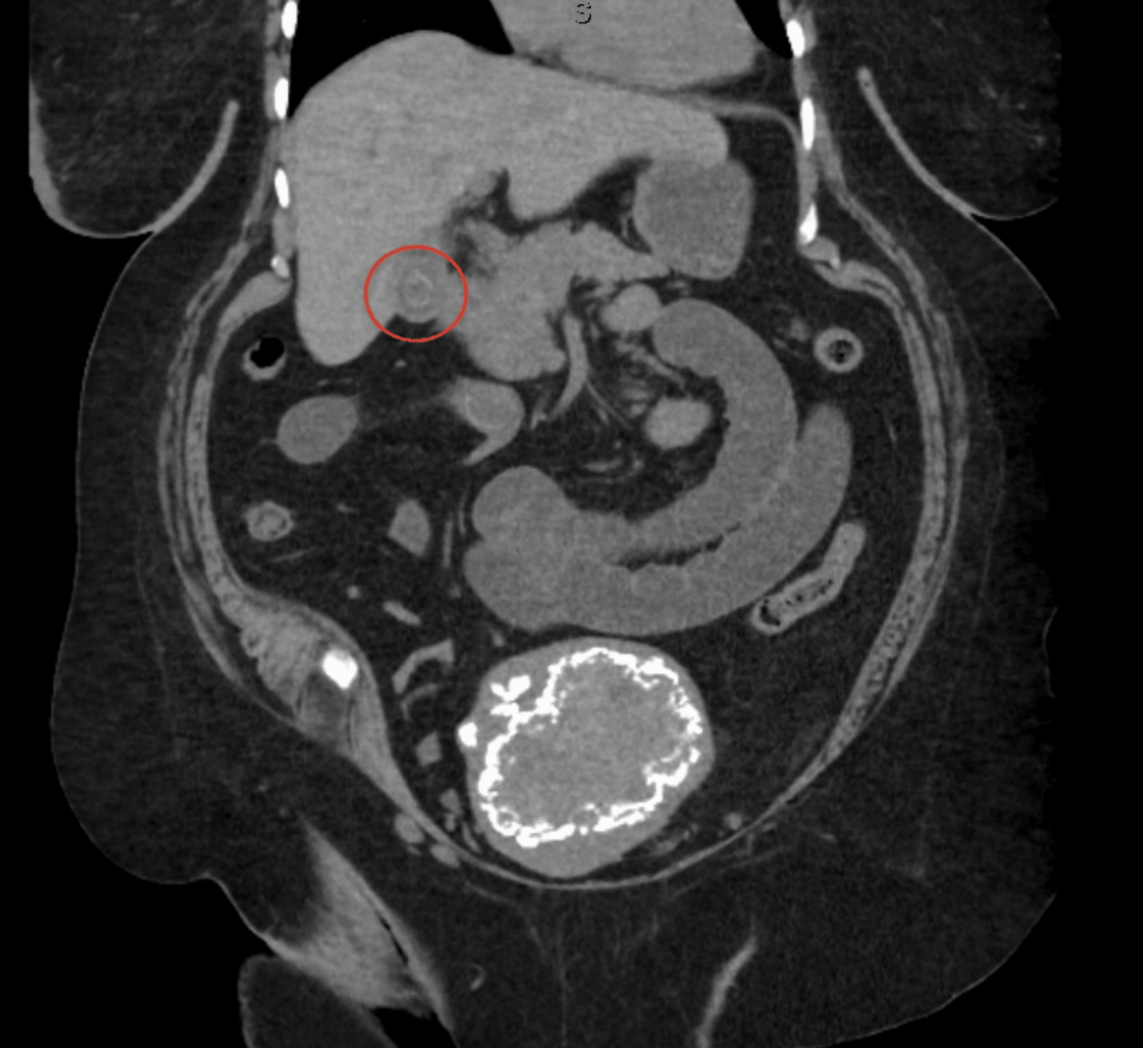
CT demonstrating cholecystoenteric fistula with a remaining stone in the gallbladder (circled in red)

A 68-year-old female patient with history of type 2 diabetes mellitus, hypertension, breast cancer, and end-stage renal disease (ESRD) on hemodialysis, obesity, but no abdominal surgeries presented to the Emergency Department (ED) with a chief complaint of abdominal pain. Her pain had begun the previous day in the morning and was accompanied by nausea, several episodes of emesis, inability to pass flatus, and inability to tolerate oral intake. She denied any previous abdominal surgeries, similar episodes in the past, and any other associated symptoms. She was hemodynamically stable and physical exam was only remarkable for generalized abdominal pain with mild distention but no peritoneal signs. Due to her ESRD, a computed tomography (CT) scan of the abdomen and pelvis without contrast was performed. It revealed a cholecystoenteric fistula involving the gallbladder fundus and first portion of the duodenum, a gallstone remaining within the bladder, and a 2.4 cm intraluminal gallstone in the small bowel, consistent with gallstone ileus (Figures 1, 2). 

**Figure 3:**
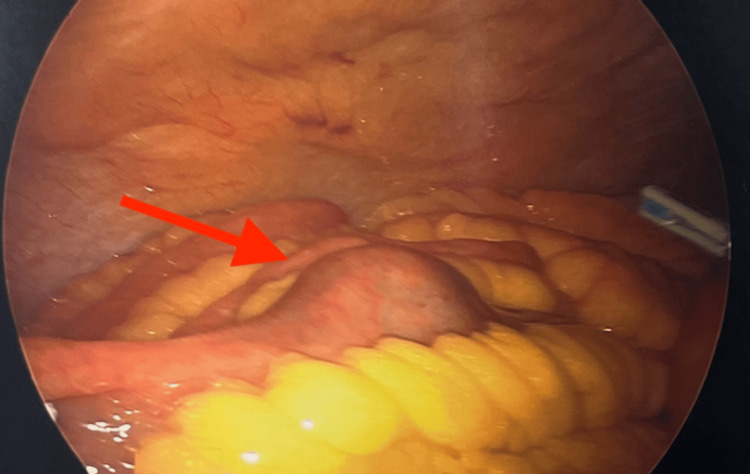
Intraoperative picture taken with Optiview demonstrating the gallstone impacted in the jejunum (red arrow)

Once the gallstone ileus was confirmed by CT, the patient was taken to the operating room (OR) for a laparoscopic-assisted enterolithotomy on the same day. Under general anesthesia, the patient was placed supine and prepped and draped in a sterile fashion. A stab incision was made in the left upper quadrant and a Veress needle was used to insufflate the abdomen. The abdomen was entered at that site using a 5 mm Optiview trocar (Endopath XCEL 5mm, Ethicon Endo-Surgery, Ohio, USA) followed by two additional 5 mm trocars under direct visualization. The bowel was examined with the 5mm Optiview from the terminal ileum to the ligament of Treitz, demonstrating the stone lodged in the distal jejunum with mild proximal bowel dilation (Figure 3).

A midline mini-laparotomy above the umbilicus was performed, an Alexis wound retractor was inserted, and the involved portion of the jejunum was delivered out of the abdomen. A longitudinal incision along the antimesenteric border was made proximal to the stone and the stone was removed. The enterotomy was closed in a transverse, two-layer fashion using 3-0 V-loc suture (3-0 V-Loc Covidien, Massachusetts USA). Before ending the procedure, the right upper quadrant was inspected. There was significant inflammation and adhesions extending throughout the liver, gallbladder and omentum. Though the residual gallstone was observed in the gallbladder on preoperative CT, we decided to not intervene on the gallbladder and the fistula at that time.

The patient had an unremarkable postoperative course. Given her moderate abdominal distention, she was kept on nil per os (NPO) and on intravenous fluids until she had return of bowel function. She was given a clear liquid diet on the second postoperative day (POD). Then she advanced to her usual renal diet on the third POD and was discharged later that evening. 

**Figure 4:**
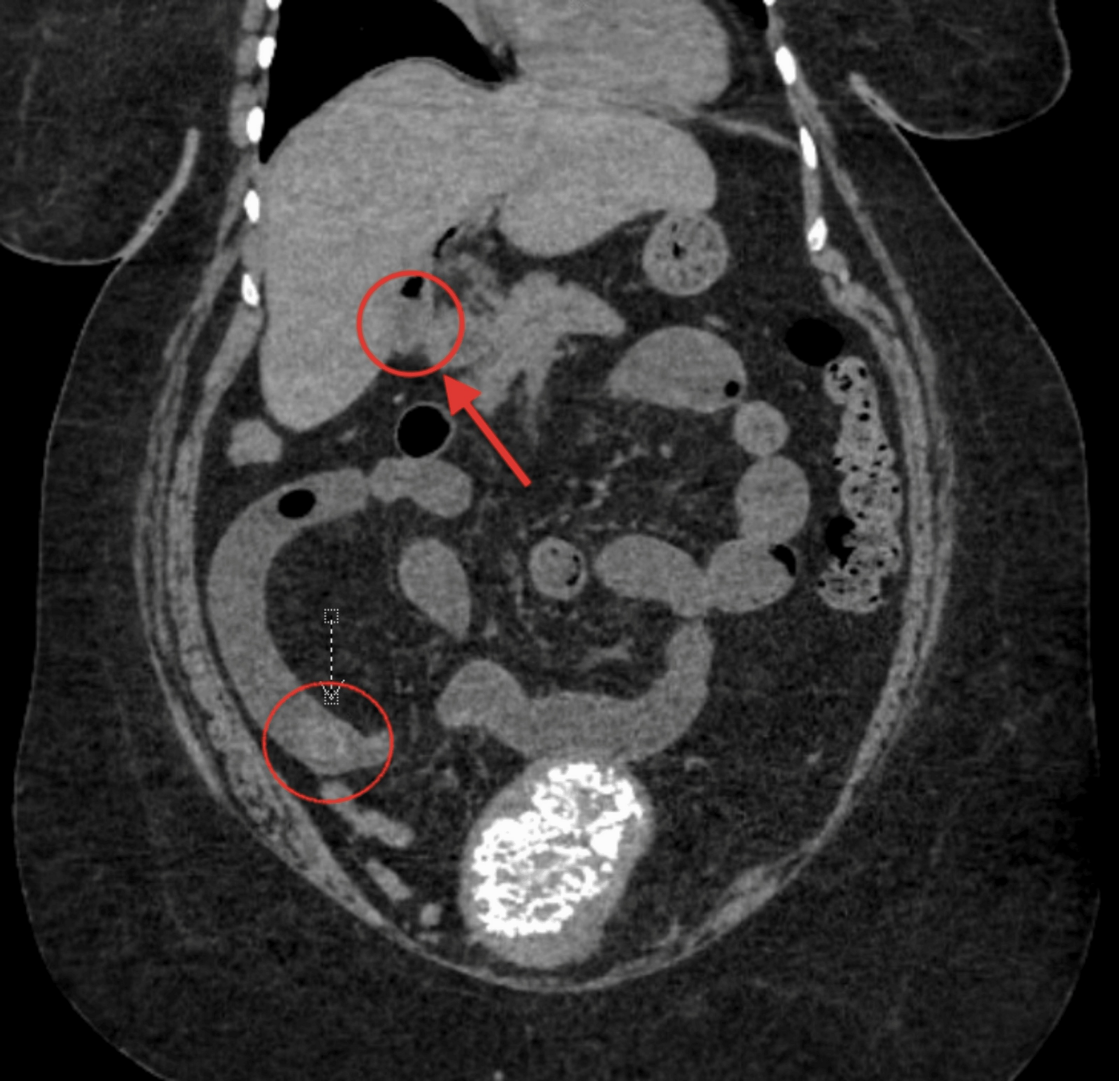
CT demonstrating an intraluminal gallstone in the distal ileum (circled in red) and a cholecystoenteric fistula (red arrow) with no remaining stones in the gallbladder

She presented to the ED again on the seventh POD with complaints of nausea, emesis, and epigastric pain that began the day after she was discharged. She denied flatus or bowel movements since symptom onset. She was hemodynamically stable with no signs of peritonitis on physical exam. Blood tests demonstrated a white blood cell count of 15,200/mm^3^. A CT of the abdomen and pelvis without contrast demonstrated a SBO secondary to a gallstone in the distal small bowel, along with the known enterobiliary fistula, but no visualized stone remaining in the gallbladder (Figure 4).

**Figure 5:**
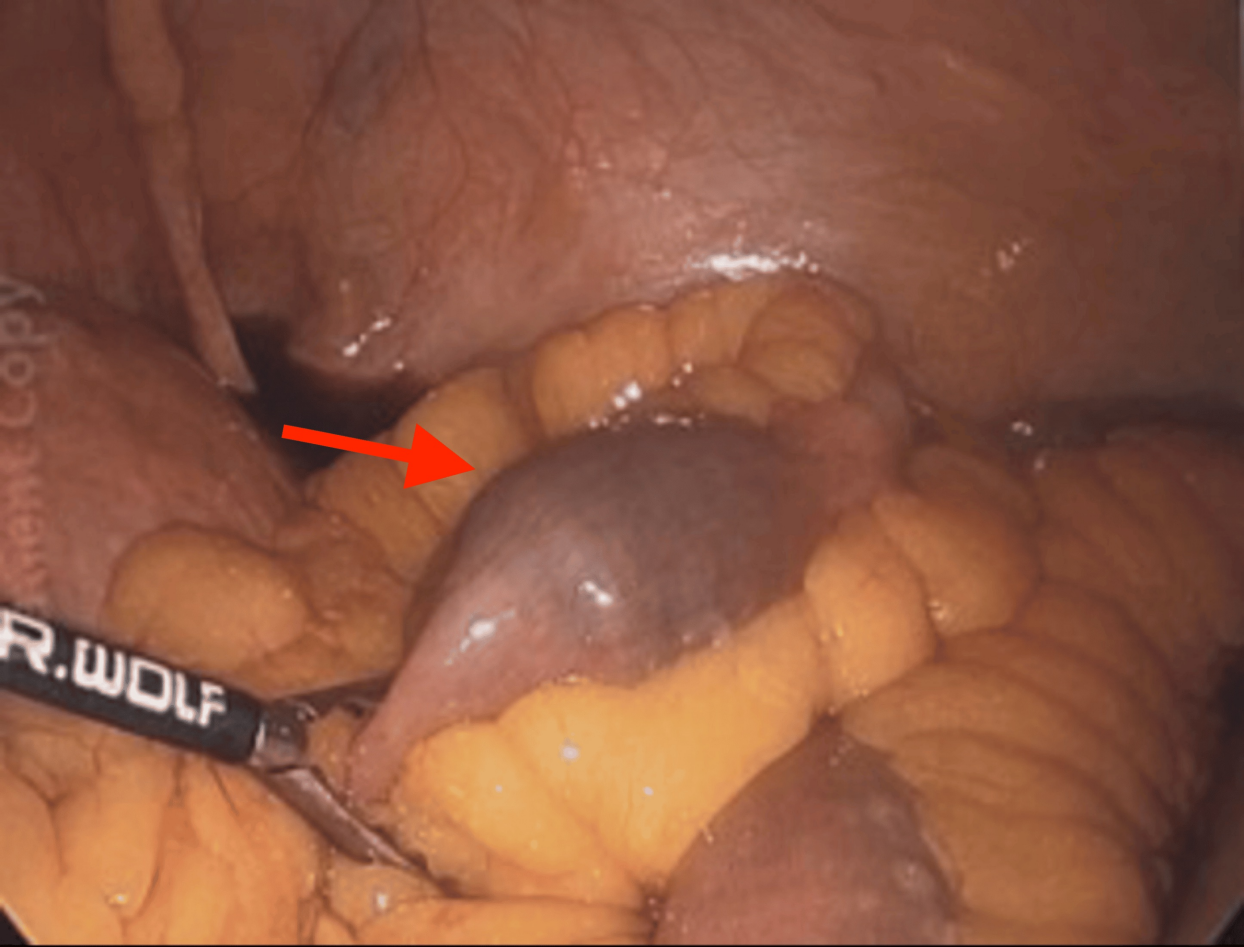
Intraoperative picture demonstrating impacted stone in the ileum (red arrow)

She was taken to the OR later that night for a diagnostic laparoscopy and laparoscopic-assisted enterolithotomy. The same incisions from her initial surgery were utilized for trocar placement. The small bowel was manually palpated and the stone was found over 20 cm distal to the previous enterotomy closure, which remained intact and was healing well (Figure 5).

**Figure 6:**
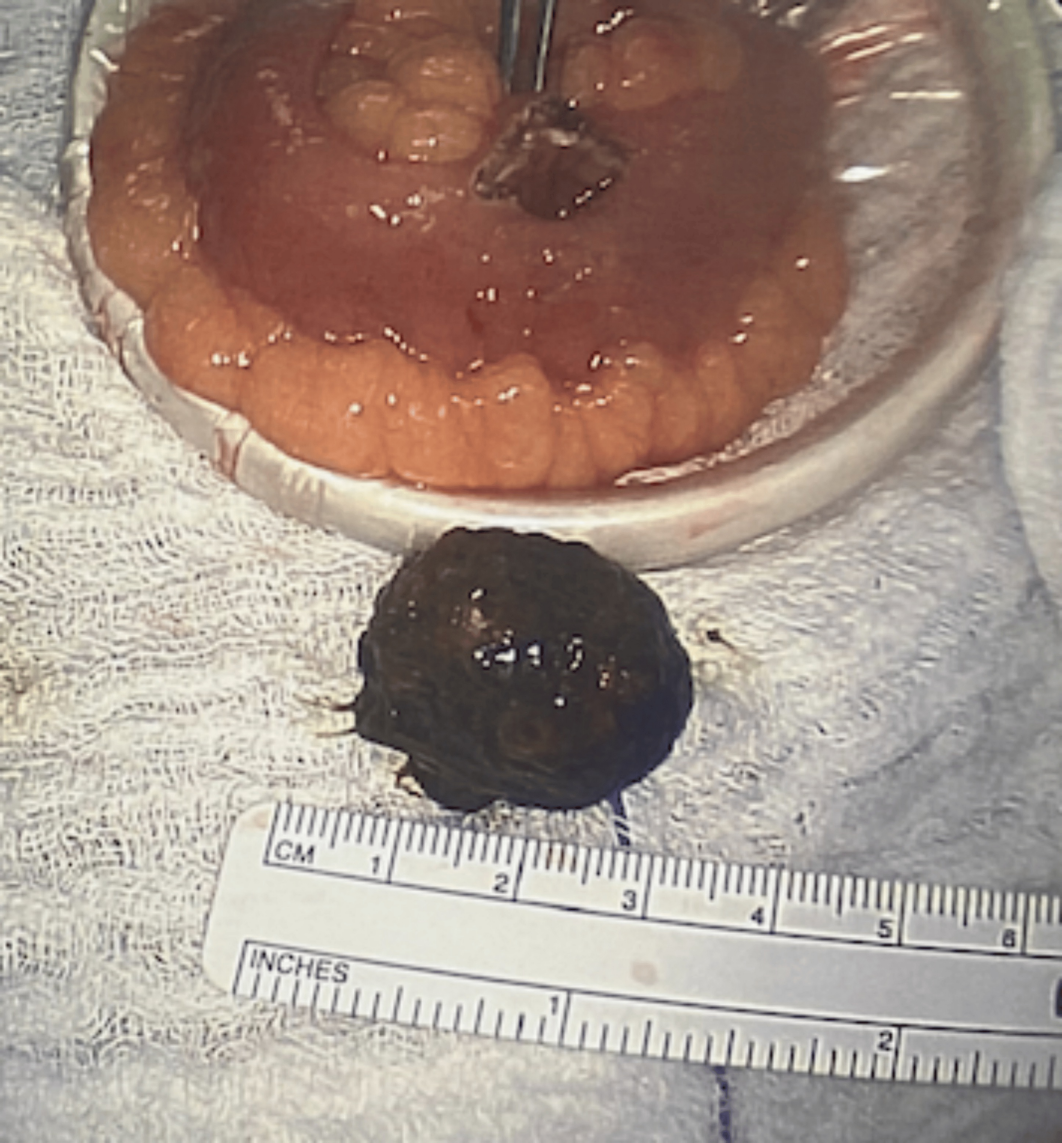
A 2.5 cm extracted gallstone and the small bowel with enterotomy

Given that the stone was significantly distal to our previous enterotomy, we performed a second enterotomy instead of a segmental resection to extract the stone and involve our previous enterotomy. A longitudinal incision was made proximally to the stone and it was removed (Figure 6).

The bowel was closed in a transverse fashion. Fortunately, the patient again had an unremarkable postoperative course. She was advanced to a renal diet on the third POD and discharged on the fifth POD, 12 days after her initial presentation. Her follow-up visit in the clinic was within two weeks of her discharge and she reported no further symptoms. 

## Discussion

Gallstone ileus is characterized by an obstruction in the gastrointestinal tract due to the passage of a gallstone through a cholecystoenteric fistula. The fistula is most frequently seen between the gallbladder and duodenum. Once inside the gastrointestinal tract, the stone travels distally and either passes spontaneously through the rectum or becomes impacted and causes an obstruction [2]. The most common site of impaction is the terminal ileum at the ileocecal valve [3]. It is a rare cause of SBO and an even rarer complication of cholelithiasis, occurring in 0.4 to 1.5% of cases [6].

Many patients presenting with gallstone ileus have a history of biliary symptoms. Clavian et al. reported rates between 27-87% of patients [8]. The clinical signs and symptoms of gallstone ileus are usually non-specific, contributing to a delay in diagnosis [9,10]. Abdominal pain, nausea, vomiting, and distension are the most frequent symptoms encountered [1]. Several diagnostic tools are available to confirm the diagnosis of gallstone ileus when suspected, mainly including plain abdominal radiograph, abdominal ultrasound, CT scan, and magnetic resonance cholangiopancreatography (MRCP) [1]. However, several studies favor CT as the gold standard in diagnosis, as it is expeditious and plays an important role in the decision-making for surgical management [5]. Yu et al. described the overall sensitivity, specificity, and accuracy of CT for diagnosing gallstone ileus to be 93%, 100%, and 99%, respectively [11]. Once the diagnosis is made, surgical intervention follows.

The extent and timing of surgery remains controversial to this day. The procedures available are simple enterolithotomy; enterolithotomy, cholecystectomy, and fistula closure (one-stage procedure); and enterolithotomy with interval cholecystectomy and fistula closure (two-stage procedure) [10]. In the majority of cases, a simple enterolithotomy is performed as it relieves the obstruction and is associated with decreased operative time, morbidity, and mortality [12]. However, not addressing the cholecystoenteric fistula can put these patients at risk for further biliary complications, such as cholangitis, gallbladder malignancy, and a recurrence of their gallstone ileus. While the recurrence of gallstone ileus is not very common (reported rates of around 5%), the underlying gallstone disease and fistula would need to be addressed in order to completely prevent a recurrence [4,6]. In their review of 1,001 cases, Reisner et al. reported an associated mortality of 16.9% with the one-stage procedure and 11.7% for the simple enterolithotomy [6]. Mir et al. showed a 4.8% mortality with simple enterolithotomy compared to 22.2% with the one-stage procedure in their 25-year subset analysis [4]. In a 2014 study, Halabi et al. also reported a significantly increased length of hospital stay and mortality rates in patients who underwent one-stage procedure [12]. Meanwhile, other studies demonstrated a significant association between one-stage procedures and complication rates [3,6]. This data indicates that enterolithotomy alone or the two-stage procedure of enterolithotomy followed by cholecystectomy and biliary fistula repair are superior to the one-stage procedure due to the lower risk of subsequent morbidity and mortality [4].

With no randomized studies in the literature, there is no current gold standard in surgical procedures for either gallstone ileus or its recurrence. However, available literature does favor simple enterolithotomy, unless there are serious contraindications [13]. Previous case reports have described successful surgical management of recurrent gallstone ileus by simple enterolithotomy, either laparoscopic-assisted or by laparotomy [14,15]. Our case describes a 68-year-old female patient who presented with recurrent gallstone ileus seven days after initial gallstone ileus, with both episodes treated with laparoscopic-assisted enterolithotomy. Our patient was able to make an expeditious and satisfactory recovery both times following surgery. For patients with recurrence, as well as increased age and comorbidities, simple enterolithotomy should be utilized whenever feasible to minimize morbidity and mortality. The advantage seen with simple enterolithotomy can be increased when performed in a minimally invasive manner. While this article demonstrates that repeat laparoscopic-assisted enterolithotomy is a safe and effective treatment option for recurrent gallstone ileus in a patient with multiple comorbidities, further research is needed to arrive at a gold standard treatment. Ultimately, surgeons will have to continue to weigh the risks and benefits utilizing the current available data and tailor the surgical management to each patient to optimize patient outcomes.

## Conclusions

This report has shown that emergency laparoscopic-assisted enterolithotomy for gallstone ileus may be complicated by recurrent ileus and highlights the importance of patient follow-up. In this case, the patient underwent laparoscopic simple enterolithotomy in two instances, seven days apart, and tolerated both procedures well with unremarkable postoperative courses. Further research comparing simple laparoscopic-assisted enterolithotomy to the one-stage and two-stage procedures is needed, especially in the setting of high-risk patients with known residual gallstones in the gallbladder, to determine the best surgical intervention. 
